# Expanding the phenotype and metabolic basis of ATP6AP2-congenital disorder of glycosylation in a Chinese patient with a novel variant c.185G>A (p.Gly62Glu)

**DOI:** 10.3389/fgene.2023.1264237

**Published:** 2023-11-22

**Authors:** Yuan Fang, Yi-Zhen Wang, Lian Chen, Xin-Bao Xie

**Affiliations:** ^1^ Department of Pathology, Anhui Provincial Children’s Hospital, Hefei, China; ^2^ Department of Pathology, Children’s Hospital of Fudan University, Shanghai, China; ^3^ Department of Hepatology, Children’s Hospital of Fudan University, Shanghai, China

**Keywords:** ATP6AP2, congenital disorders of glycosylation, X-linked, hereditary, cirrhosis

## Abstract

**Background:** A rare X-linked hereditary condition known as ATP6AP2-congenital disorder of glycosylation (ATP6AP2-CDG) is caused by pathogenic variants in *ATP6AP2*, resulting in autophagic misregulation with reduced siganling of mammalian target of rapamycin (mTOR) that clinically presents with aberrant protein glycosylation, hepatosteatosis, immunodeficiency, cutis laxa, and psychomotor dysfunction. To date, only two missense mutations have been reported in three patients from two unrelated families.

**Methods:** In order to extend the profiles of phenotype and genotype associated with ATP6AP2-CDG, we assessed the clinical history, whole exome sequencing (WES), and liver histology as well as immunohistochemistry in a Chinese patient, and performed quantitative real-time polymerase chain reaction (qRT-PCR), Western blotting and untargeted metabolomics in genetic exogenously constructed cells.

**Results:** The 11-month-old Chinese boy presented with recurrent jaundice, cutis laxa, cirrhosis, growth retardation, coagulopathy, anemia, and cardiomegaly, and underwent liver transplantation. A novel mutation, c.185G>A (p.Gly62Glu), was identified in exon 3 of *ATP6AP2*. The expression of ATP6AP2 was observed to remain unchanged in the liver sample of the patient as well as in HEK293T cells harboring the p.Gly62Glu. This missense mutation was found to dysregulate autophagy and mTOR signaling. Moreover, metabolomics analysis revealed that the exogenously introduced Gly62Glu mutant resulted in the downregulation of numerous metabolites involved in lipid metabolism pathway.

**Conclusion:** This study may enable a more detailed exploration of its precise pathogenesis and potential therapeutic interventions.

## Introduction

There is a group of Mendelian diseases, congenital disorders of glycosylation (CDGs), affecting the glycosylation of proteins or lipids, which is a post-translational modification to acquire complete biological function. Defective glycosylation involved in CDGs includes disruption of N-linked glycosylation, O-linked glycosylation, glycosylphosphatidylinositol anchor biosynthesis, multiple glycosylation pathways, and others (Ondruskova et al., 2021). According to the isoelectrofocusing (IEF) of serum transferrin, CDGs are classified into type Ⅰ and type Ⅱ patterns. While a type II pattern (increased trisialo- and monosialotransferrin) suggests a remodeling problem, a type I pattern (decreased tetrasialotransferrin, increased disialo- and asialotransferrin) indicates an assembly flaw or a defect in the transfer to the peptide chain (Peanne et al., 2018).

Since the first CDG was reported in 1980, more than 170 types of CDG have been identified with the development of genetic testing approaches. Recently, a new subtype ATP6AP2-CDG (OMIM: 301045) was reported in three patients from two unrelated families (Rujano et al., 2017). Patients presented with hepatosteatosis, immunodeficiency, cutis laxa, and psychomotor impairment. Serum transferrin glycosylation profiles were consistent with a type Ⅱ transferrin IEF pattern. *In vitro* and *in vivo* experiments validated the mutations leading to the hypoglycosylation in a pathogenetic cascade, including degradation of misfolded proteins in the ER, impairment of interaction with the assembly factor ATP6AP1, defective lysosomal acidification, downregulation of mammalian target of rapamycin (mTOR) signaling, and induction of autophagy defection.

In this study, we identified the first Chinese patient diagnosed with ATP6AP2-CDG, presenting with recurrent jaundice, cutis laxa, cirrhosis, growth retardation, coagulopathy, anemia, and cardiomegaly. Whole exome sequencing (WES) revealed a hemizygous variant, the c.185G>A (p.Gly62Glu) mutation in exon 3 of *ATP6AP2*, which has not been previously reported. The expression of ATP6AP2 in the liver was normal as well as in HEK293T cells harboring p.Gly62Glu. Furthermore, in HEK293T cells, the missense mutation dysregulated autophagy and mTOR signaling. Metabolomics analysis in HEK293T cells indicated that in the exogenously introduced Gly62Glu mutant, most metabolites were downregulated and involved in the pathway of lipid metabolism.

## Materials and methods

### Subject

The clinical data of a patient with ATP6AP2-CDG was gathered at the Children’s Hospital of Fudan University. This study was approved by the Ethics Committee of Children’s Hospital of Fudan University (No. FELL-2020-402). Any potentially identifiable photographs or data were published with the parents’ written informed consent.

### Liver sample and immunohistochemistry

The material was removed from the liver transplant procedure, fixed in 10% buffered formalin, dried in varying degrees of ethyl alcohol, and then embedded in paraffin. Hematoxylin and eosin (HE) staining was followed by periodic acid Schiff (PAS) with and without diastase, Masson, reticulin, copper, and iron special staining.

Additional 4 µm sections were deparaffinized, rehydrated, and pretreated with 3% H_2_O_2_ to eliminate endogenous peroxidase activity. Besides, the sections were heat-mediated antigen-retrieved with EDTA (pH 9) or citrate buffer (pH 6) before immunohistochemical staining was undertaken. A variety of antibodies were used as primary antibodies, including ATP6AP2 (purchased from www.ptgcn.com, catalog number 10926-1-AP, 1:50 dilution), LC3A/B (purchased from www.affbiotech.com, catalog number AF5402, 1:100 dilution), and mTOR (purchased from www.affbiotech.com, catalog number AF6308, 1:100 dilution). After incubating overnight at 4°C, the sections were incubated at room temperature for an hour with the general secondary antibody. Then, the sections were developed with DAB and counterstained with hematoxylin. To serve as a control, we used a normal liver sample donated by a surgical patient.

### Genetic analysis

We collected the patient’s and his parents’ EDTA-anticoagulated whole blood specimens to perform WES004 (Human All Exon *V4*) using the Illumina HiSeq X ten platform by MyGenostics (a commercial genetic testing company). Using the TruSeq DNA Library Preparation Kit, DNA libraries were created. The raw data were aligned to the reference human genome (GRCh37/hg19). For variation calling to compile single nucleotide variations (SNVs) and indels, the Genome Analysis Toolkit (GATK) (https://gatk.broadinstitute.org) was applied. The annotation and interpretation processes used ANNOVAR software and the Enliven^®^ Variants Annotation Interpretation System. Data were filtered in 1,000 Genome (www.1000 genomes.org), NHLBI Exome Sequencing Project (ESP6500) (https://esp.gs.washington.edu), Genome Aggregation Database (gnomAD) (https://gnomad.broadinstitute.org), dbSNP152 (https://www.ncbi.nlm.nih.gov/snp), and Exome Aggregation Consortium (ExAC) (http://exac.broadinstitute.org). In order to score the genetic variants and further compare them, damage prediction was conducted using Combined Annotation Dependent Depletion (CADD) (https://cadd.gs.washington.edu) and Mutation Significance Cutoff (MSC) (https://lab.rockefeller.edu/casanova/MSC). With a 99% confidence level and HGMD and ClinVar as the database sources, the MSC server was deployed to CADD, PolyPhen 2 (http://genetics.bwh.harvard.edu/pph2), and SIFT (https://sift.bii.a-star.edu.sg/www/SIFT_indels2.html). For analysis based on variant-disease and gene-disease relationships, Genomics England PanelApp, a crowdsourcing tool, was used (https://panelapp.genomicsengland.co.uk). The HGMD database, Online Mendelian Inheritance in Man (OMIM), and Human Phenotype Ontology (HPO) were utilized to correlate phenotype descriptions with variant and gene prioritization results. Genetic variants were categorized into five categories by the American College of Medical Genetics and Genomics (ACMG): pathogenic, likely pathogenic, variant of uncertain significance (VUS), likely benign, and benign. Following that, a VUS of *ATP6AP2* was discovered in the patient with the transcript NM_005765, the mutation site was confirmed by Sanger sequencing in his parents.

MutationTaster (http://www.mutationtaster.org) predicted the toxicity of the amino acid shift brought on by the gene mutation. In addition, the conservation of the mutation site was analyzed among multiple species. We modeled the protein using SWISS-model (https://www.swissmodel.expasy.org) with the UniProtKB code O75787, and PyMol (http://www.pymol.org) was used to analyze and visualize the altered structure.

### Cell culture and plasmids transfection

HEK293T cells were cultured in high-glucose DMEM (Gibco) supplemented with 10% fetal bovine serum and 1% penicillin/streptomycin at 37°C under 5% CO_2_. The cDNA of *ATP6AP2* was generated by gene synthesis (www.generalbiol.com) and cloned into pcDNA3.1 with an N-terminal FLAG-tagged version. The original plasmid was used as a template to introduce the site mutation ATP6AP2^Gly62Glu^ by homologous recombination. All constructs were confirmed by DNA sequencing. The plasmids were chemically transformed into competent DH5α E. coli (TIANGEN). Individual colonies were selected, and plasmid DNAs were extracted using OMEGA Endo-Free Plasmid Midi Kit D6915. Then, Beyotime Lipo8000™ Transfection Reagent was used for plasmid DNA transfection following the manufacturer’s instructions.

### RNA extraction, reverse transcription, and quantitative real-time PCR

Total RNA was extracted using AG RNAex Pro Reagent. Reverse transcription of the total RNA into cDNA was carried out using Evo M-MLV RT Premix (Accurate Biotechnology). The relative expression of endogenous, wild-type, and mutant *ATP6AP2* was determined by quantitative real-time polymerase chain reaction (qRT-PCR) using SYBR Green Premix Pro Taq HS Kit (Accurate Biotechnology) on a Bio-Rad system. The forward primer sequence is 5′CTG​CAT​TGT​CCA​TGG​GCT​TC3′, and the reverse primer sequence is 5′AAC​AGG​TTA​CCC​ACT​GCG​AG3′.

### Western blotting

Cells were harvested in 100 µL RIPA buffer supplemented with 1 µL phenylmethanesulfonyl fluoride lysed on ice for 30 min. The lysates were then centrifuged at 12,000 rpm for 20 min at 4°C. The supernatant was measured for the protein concentration using the Pierce BCA Protein Assay Kit (Thermo Fisher Scientific) and was denatured with 1× SDS-PAGE Sample Loading Buffer (Beyotime) at 100°C for 10 min. We measured 10 µg total protein of each aliquot, and it was analyzed by SDS-PAGE and immunoblotted to a hydrophobic polyvinylidene fluoride membrane. After 1 h of milk blocking, the membranes were incubated with primary antibodies at 4°C overnight followed by second antibodies. Bands signal was detected by Tanon 5,200 automatic chemiluminescence image analysis system, and the relative expression was calculated by ImageJ software.

The following antibodies were used: ATP6AP2 (purchased from www.ptgcn.com, catalog number 10926-1-AP, 1:1,000 dilution), LC3A/B (purchased from www.affbiotech.com, catalog number AF5402, 1:1,000 dilution), mTOR (purchased from www.affbiotech.com, catalog number AF6308, 1:1,000 dilution), FLAG tag (purchased from www.huabio.cn, catalog number HA601080, 1:5,000 dilution) and GAPDH (purchased from Zsbio, catalog number TA-08, 1:2000 dilution). HRP-labeled Goat Anti-Rabbit (purchased from www.beyotime.com, catalog number A0208, 1:20000 dilution) and HRP-labeled Goat Anti-Mouse IgG (purchased from www.beyotime.com, catalog number A0216, 1:20000 dilution) were used as second antibodies.

### Untargeted metabolomics

HEK293T cells harboring ATP6AP2^WT^ and ATP6AP2^Gly62Glu^ were cultured in 10 cm dishes. Each group of six samples was extracted with extractant containing internal standard (methanol:acetonitrile:water = 2:2:1, V/V/V) for metabolite extraction. Metabolomic data analyses were conducted using non-targeted metabolomic profiling by Biomarker Co., Ltd. The Waters Acquity I-Class PLUS ultra-high performance liquid tandem mass spectrometer and the Waters Xevo G2-XS QTof high-resolution mass spectrometer made up the LC/MS equipment used for the metabolomics investigation. The column used was purchased from Waters Acquity UPLC HSS T3 column (1.8 um 2.1*100 mm). Based on the Progenesis QI software’s online METLIN database and Biomark’s own self-built library for identification, the raw data collected using MassLynx V4.2 were processed by Progenesis QI software for peak extraction, peak alignment, and other data processing operations. At the same time, theoretical fragment identification and mass deviation were both within 100 ppm. The follow-up study was carried out after the first peak area data was normalized with the total peak area. In order to assess the repeatability of the samples within the group and the quality control samples, principal component analysis and Spearman correlation analysis were utilized. The KEGG, HMDB, and lipid mapping databases were examined for classification and route information pertaining to the discovered chemicals. The OPLS-DA modeling was carried out using the R language package, and the model’s dependability was examined using 200 permutations.

### Statistical analysis

Data were analyzed using SPSS 20.0 and statistical plotting was performed using GraphPad Prism 8.0 software. Metric data were expressed in (mean ± standard deviation, ± s). A parametric *t*-test was used for comparisons of metric data that followed a normal distribution, and a nonparametric *t*-test was used when the normal distribution was not followed. Statistics were considered significant for *p*-values under 0.05.

## Results

### Clinical description

The boy was born to healthy non-consanguineous parents and was delivered by a full-term cesarean section with a birth weight of 3.5 kg. From 3 days to 2 months age, he was hospitalized three times for jaundice. During routine blood tests, remarkably elevated total bilirubin, direct bilirubin, alanine aminotransferase, aspartate aminotransferase, γ-glutamyl transferase, total cholesterol, low-density lipoprotein (LDL) cholesterol, international normalized ratio, prolonged prothrombin time, and decreased serum ceruloplasmin were noticed. There was no hepatosplenomegaly, no significant anemia, no elevated reticulocytes, and no hemolytic manifestations. The patient was discharged after the condition improved, followed by treatment with ursodeoxycholic acid (UDCA), compound glycyrrhizin tablets, and traditional Chinese medicine (details are not available). However, the hyperbilirubinemia, hypertransaminasemia, coagulopathy, and decreased serum ceruloplasmin persisted, and massive ascites emerged during the approximate 9-month follow-up. Besides, alpha-fetoprotein (AFP) (1,395 ng/mL, reference range 0–8.1 ng/mL) was elevated at 10 months of age, which might result from his young age and liver impairment.

**FIGURE 1:**
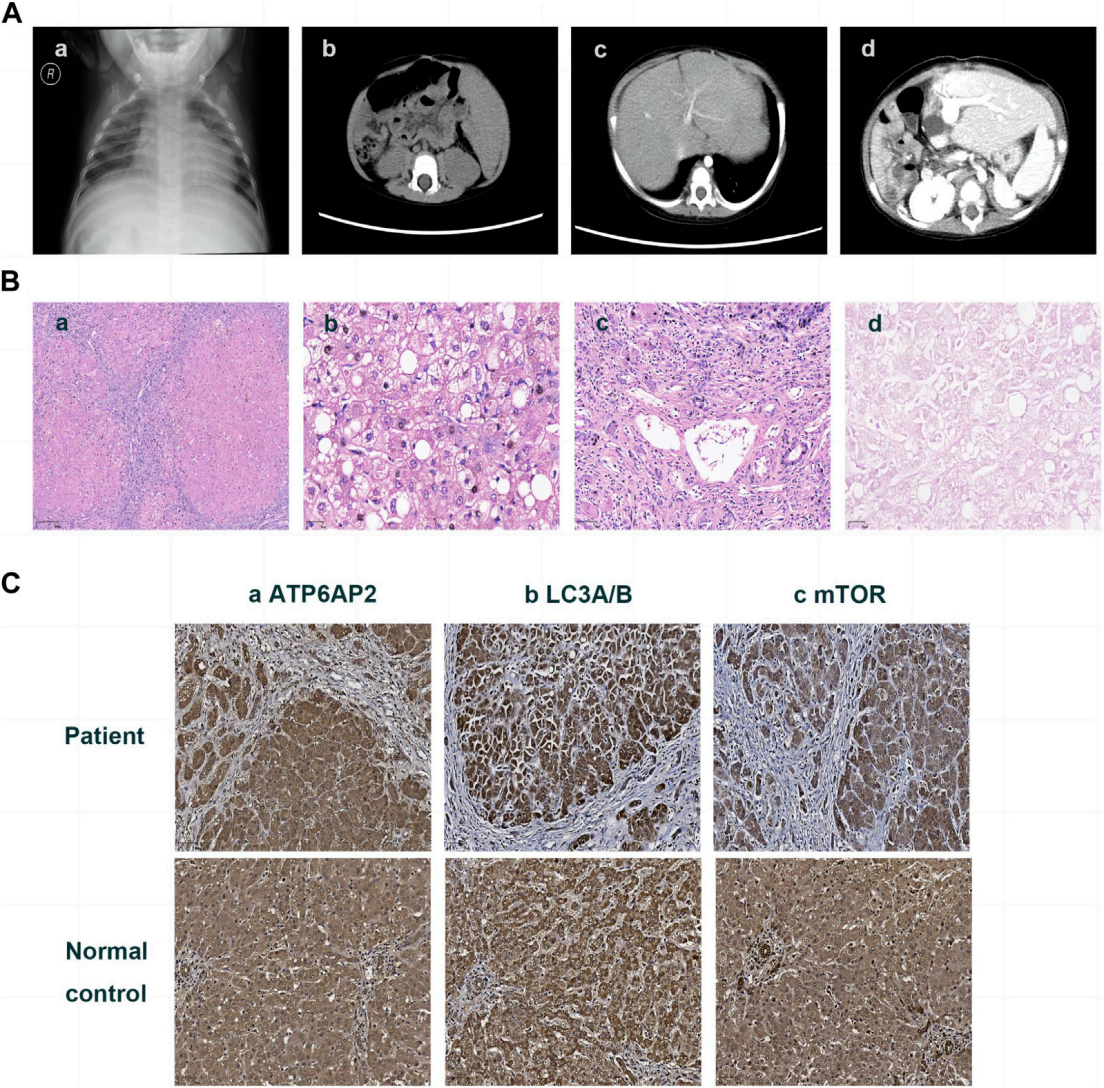
Imaging and pathologic findings of the patient. An enlarged heart shadow was displayed in X-ray **(A)**-**a**. CT showed multiple hepatic hypointensive foci and splenomegaly **(A)**-**b** and revealed portal hypertension and lateral branch circulation formation as well **(A)**-**c**. Portal anastomotic stenosis was present after liver transplantation **(A)**-**d**. HE staining showed the distorted architecture of liver lobules and the morphology of pseudo-lobules **(B)**-**a** ×40, hepatic cells with finely granular cytoplasm and clear cell membrane, along with obvious vesicular steatosis in the portal area **(B)**-**b** ×200, and lymphocytic infiltration in the portal area **(B)**-**c** 100. PAS staining identified various sizes of vacuoles in partial hepatocytes **(B)**-**d** ×200. Immunohistochemistry on the liver showed hepatocytes positive for ATP6AP2 **(C)**-**a** ×100, LC3A/B **(C)**-**b** ×100 and mTOR **(C)**-**c** ×100, without obvious differences in expression compared to the normal control.

Then the 11-month-old boy was admitted to our hospital for further therapy. Physical examination showed that his height was 70 cm (<3rd percentile by the WHO standard) and weight was 7.4 kg (<3rd percentile). There was mild yellowing of the sclera and skin throughout the body without rash or bleeding points. Cutis laxa was noticed in the patient. There was an II/VI murmur of the heart at the left sternal border. Abdominal distention was present. The liver was located 3.5–4 cm below the xiphoid process, with a tough texture. The spleen was located reaching the iliac fossa below the left costal, and right to the midline, with a diameter of 9–10 cm. No anomalies were discovered during the nervous system evaluation. Echocardiography and X-ray revealed cardiomegaly (Figure 1A-a). Normal cardiac function was assessed since a normal left ventricular ejection fraction without manifestations of left or right ventricular insufficiency. The patient did not present with hypertrophic cardiomyopathy as well. Abdominal ultrasonography indicated multiple hepatic focal lesions and splenomegaly; the former presented as an enhancement on computed tomography (CT) (Figure 1A-b). CT showed portal hypertension and lateral branch circulation formation as well (Figure 1A-c). The development screening test (DST) showed growth retardation of the patient. The majority of biochemical liver test results were abnormal, including low albumin, elevated aspartate aminotransferase, γ-glutamyl transferase, alkaline phosphatase, creatine kinase isoenzyme, total bilirubin, direct bilirubin, and total bile acid. Prolonged activated partial thromboplastin time and prothrombin time, increased international normalized ratio and D-dimer, and decreased fibrinogen were present. Complete blood count demonstrated anemia and thrombocytopenia without immunodeficiency. The patient’s primary laboratory investigations at various stages are summarized in Table 1. Symptomatic treatment was then carried out by albumin and fibrinogen infusion, oral administration of D-galactose, UDCA, and vitamin D/AD/E/K1 supplements. Among them, D-galactose was administered to correct abnormal glycosylation metabolic pathway in purpose. Due to chronic liver failure with life-threatening decompensated cirrhosis and an increased risk of transplantation as his physical condition deteriorated, the patient underwent living-related liver (donated by his mother) transplantation at that time. At subsequent follow-up, ultrasound and CT showed portal anastomotic stenosis (Figure 1A-d). Liver function parameters gradually normalized, and cutis laxa also improved post transplantation. Tacrolimus was given to control the graft versus host reaction. The patient has been followed through 1 year postoperatively and recovered well.

**TABLE 1:** Predominant changes in laboratory investigations of the current patient.

Age (m, month)		2 m	4 m	10 m	11 m	12 m	13 m	14 m	16 m	17 m	20 m	21 m
Serum biochemistry (reference range)	Albumin (35–55 g/L)	NA	NA	NA	23.49	43.99	26.84	28.20	37.95	39.96	35.71	37.23
	Globulin (20–30 g/L)	NA	NA	NA	NA	24.41	16.56	NA	NA	16.10	16.49	17.07
	Alanine aminotransferase (0–40 IU/L)	68.72	26.00	47.00	NA	21.00	44.40	46.91	15.69	32.76	37.91	15.22
	Aspartate aminotransferase (0–40 IU/L)	166.13	112.00	241.00	161.00	61.00	131.25	139.61	27.07	58.28	75.81	37.14
	γ-glutamyl transferase (7–50 IU/L)	125.18	180.00	409.00	NA	48.00	40.73	31.94	27.82	15.93	22.54	20.31
	Total bilirubin (5.1–17.1 μmol/L)	233.85	88.00	190.00	71.00	107.30	119.80	165.10	9.20	15.50	11.30	8.30
	Direct bilirubin (0–6 μmol/L)	13.88	4.80	44.90	34.99	37.10	54.00	89.20	3.30	6.30	3.70	3.60
	Total bile acid (0–10 μmol/L)	NA	NA	NA	NA	15.70	123.30	371.30	2.90	5.20	7.10	11.70
	Alkaline phosphatase (42–383 IU/L)	NA	NA	NA	NA	593.00	1,100.88	NA	NA	91.07	390.66	245.09
	Ammonia (10–47 μmol/L)	NA	NA	NA	NA	66.00	119.00	110.00	92.00	105.00	NA	NA
	Total cholesterol (3.1–5.2 mmol/L)	7.24	NA	NA	2.87	1.46	1.55	NA	NA	NA	NA	NA
	LDL-cholesterol (1.30–3.90 mmol/L)	5.12	NA	NA	2.04	NA	NA	NA	NA	NA	NA	NA
	HDL-cholesterol (0.91–2.05 mmol/L)	1.53	NA	NA	1.01	NA	NA	NA	NA	NA	NA	NA
	Triglyceride (0.56–1.70 mmol/L)	1.30	NA	NA	0.68	0.39	0.44	NA	NA	NA	NA	NA
	Ceruloplasmin (0.22–0.58 g/L)	<0.02	NA	NA	0.08	0.17	NA	NA	NA	NA	NA	NA
Blood coagulation profiles (reference range)	Activated partial thromboplastin time (28.0–44.5 s)	NA	NA	NA	NA	62.40	71.40	77.80	47.50	NA	NA	NA
	D-dimer (0–0.3 mg/L)	NA	NA	NA	NA	2.09	1.00	0.97	1.95	NA	NA	NA
	Fibrinogen (2–4 g/L)	NA	NA	NA	0.97	1.57	0.76	0.95	2.77	NA	NA	NA
	International normalized ratio (0.8–1.2)	1.63	NA	NA	2.10	2.10	3.00	3.20	1.75	NA	NA	NA
	Prothrombin time (12.0–14.8 s)	30.60	NA	NA	NA	24.00	31.80	33.50	20.00	NA	NA	NA
	Prothrombin time activity (80%–100%)	NA	NA	NA	30.40	35.00	24.00	NA	NA	NA	NA	NA
Complete blood count (reference range)	Red blood cell (3.5–5.6 × 10^12^/L)	NA	NA	NA	NA	2.11	2.45	2.41	4.09	3.76	3.99	3.87
	White blood cell (5.6–14.5 × 10^9^/L)	NA	NA	NA	8.07	5.19	6.29	7.17	5.71	5.27	8.46	8.85
	Hemoglobin (99–196 g/L)	NA	NA	NA	79	67	72	71	NA	102	97	93
	Platelet (203–653 × 10^9^/L)	NA	NA	NA	69	57	78	66	207	145	129	128
	Reticulocyte percentage (0.82%–2.25%)	NA	NA	NA	NA	4.0	5.1	NA	3.5	NA	4.0	4.9

NA: not available.

### Histology and immunohistochemistry of the liver

The liver specimen resected from transplantation surgery showed distorted architecture of liver lobules and morphology of pseudo-lobule (Figure 1B-a). High power displayed hepatic cells with finely granular cytoplasm and clear cell membrane, as well as obvious vesicular steatosis in the portal area rather than around the central vein (Figure 1B-b). The portal area was enlarged with infiltration of a lot of lymphocytes (Figure 1B-c). PAS staining identified various sizes of vacuoles in the hepatocytes with steatosis (Figure 1B-d); while staining for PAS with diastase, copper and iron were negative. The liver lobules were divided by hyperplastic fibrous tissue in the portal area, which created pseudo-lobules and bridging-like fibrosis that were identified by Masson staining. Reticulin staining revealed preserved reticular scaffold structure. According to the Ishak staging system (Ishak. et al., 1995), it was diagnosed as stage 6 cirrhosis.

Immunohistochemical staining of the patient’s liver showed a cytoplasmic positive pattern in hepatocytes for ATP6AP2 (Figure 1C-a), as well as LC3A/B (Figure 1C-b) and mTOR (Figure 1C-c). Compared with the healthy control, there were no obvious alterations observed.

### A novel germline mutation in *ATP6AP2*

**FIGURE 2:**
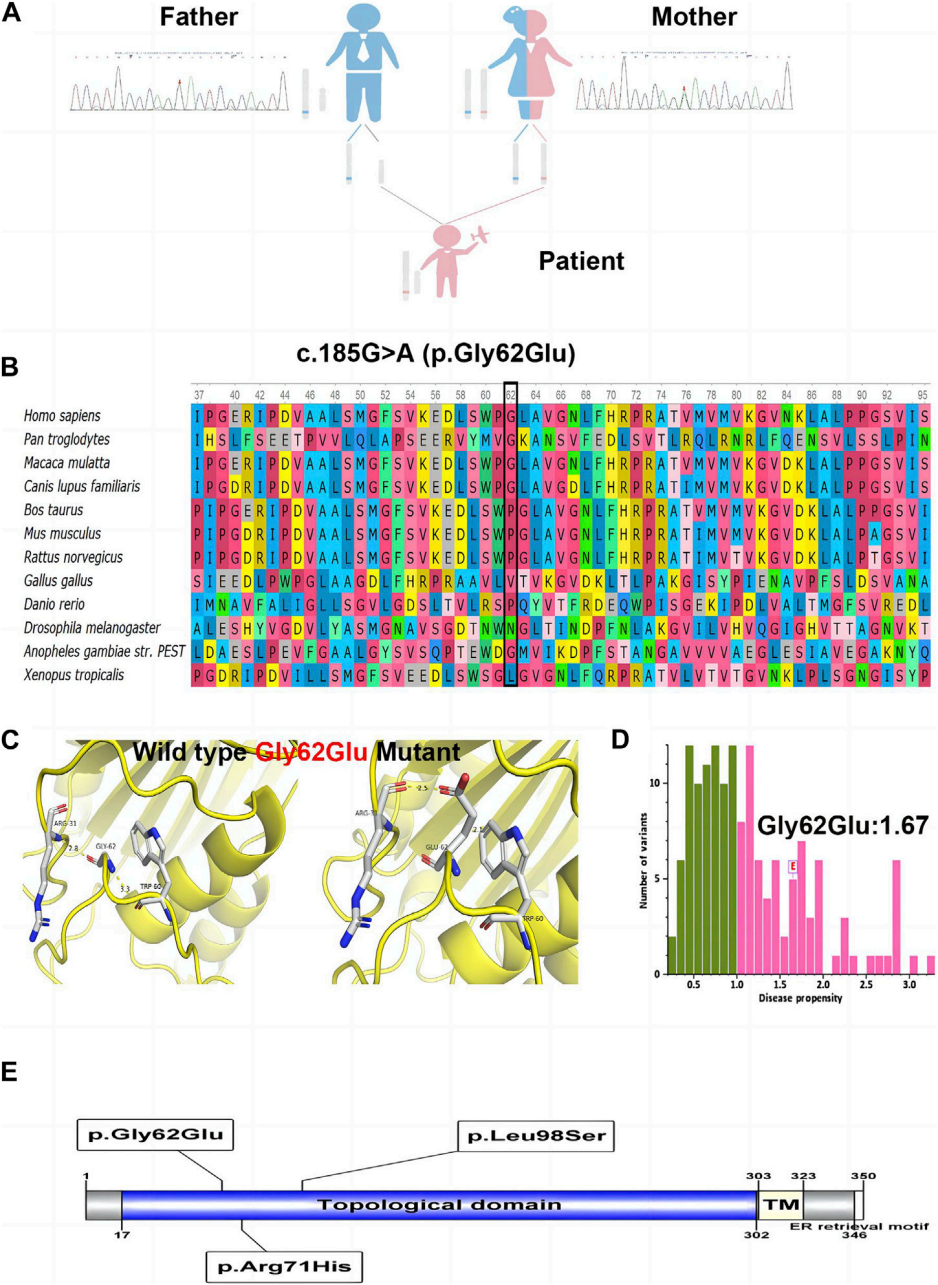
The identified c.185G>A (p.Gly62Glu) in *ATP6AP2*. Genetic genealogy diagram and Sanger sequencing confirmation in the parents **(A)**. Conservation status of amino acid residue of the variant across various species **(B)**. Wild and mutated type of the p. Gly62Glu variant compared by PyMol **(C)**. The variant of interest, Gly62Glu, had a disease propensity of 1.67, and was marked on the histogram with a boxed label **(D)**. Illustration of ATP6AP2 protein domains, location of amino acid changes of the reported variants in CDG so far **(E)**.

WES identified a hemizygous variation c.185G>A in *ATP6AP2* exon 3. The c.185G>A was inherited from the healthy mother (Figure 2A), leading to a change of glycine to glutamic acid at the amino acid position 62 (p.Gly62Glu), which was not reported in the HGMD. The amino acid residue of the site mutation was fairly well conserved (Figure 2B), which might be important for the protein’s function. The CADD score of c.185G>A was 27.9, predicted as deleterious. According to the ACMG guidelines, the variant was initially determined as clinically insignificant (uncertain) PM2: the mutation frequency was 0 in the general population database; there were no reports in the literature; comprehensive bioinformatic predictions indicated that it was harmful; and it was verified through family analysis. The Human Genome Variation Society (HGVS; http://www.hgvs.org/varnomen) guidelines served as the foundation for the nomenclature of variants. Wild-type and mutated ATP6AP2 were modeled by PyMol (http://www.pymol.org), which showed that the non-polar aliphatic amino acid glycine was substituted by acidic amino acid glutamic acid with altered polar contact with Arg31 and Trp60 (Figure 2C). The absence of a side chain in the wild-type glycine increases the flexibility of the protein at this site. The variant residue glutamic acid has a negatively charged side chain, making it hydrophilic and resulting in it preferring the surface of the protein to its interior. The glycine to glutamic acid residue change had a high “disease propensity” value of 1.67 (Figure 2D). Figure 2E illustrates the ATP6AP2 protein domains and location of amino acid changes of the reported variants in CDG so far.

### Expression of ATP6AP2^Gly62Glu^ and regulation of autophagy and mTOR signaling

**FIGURE 3:**
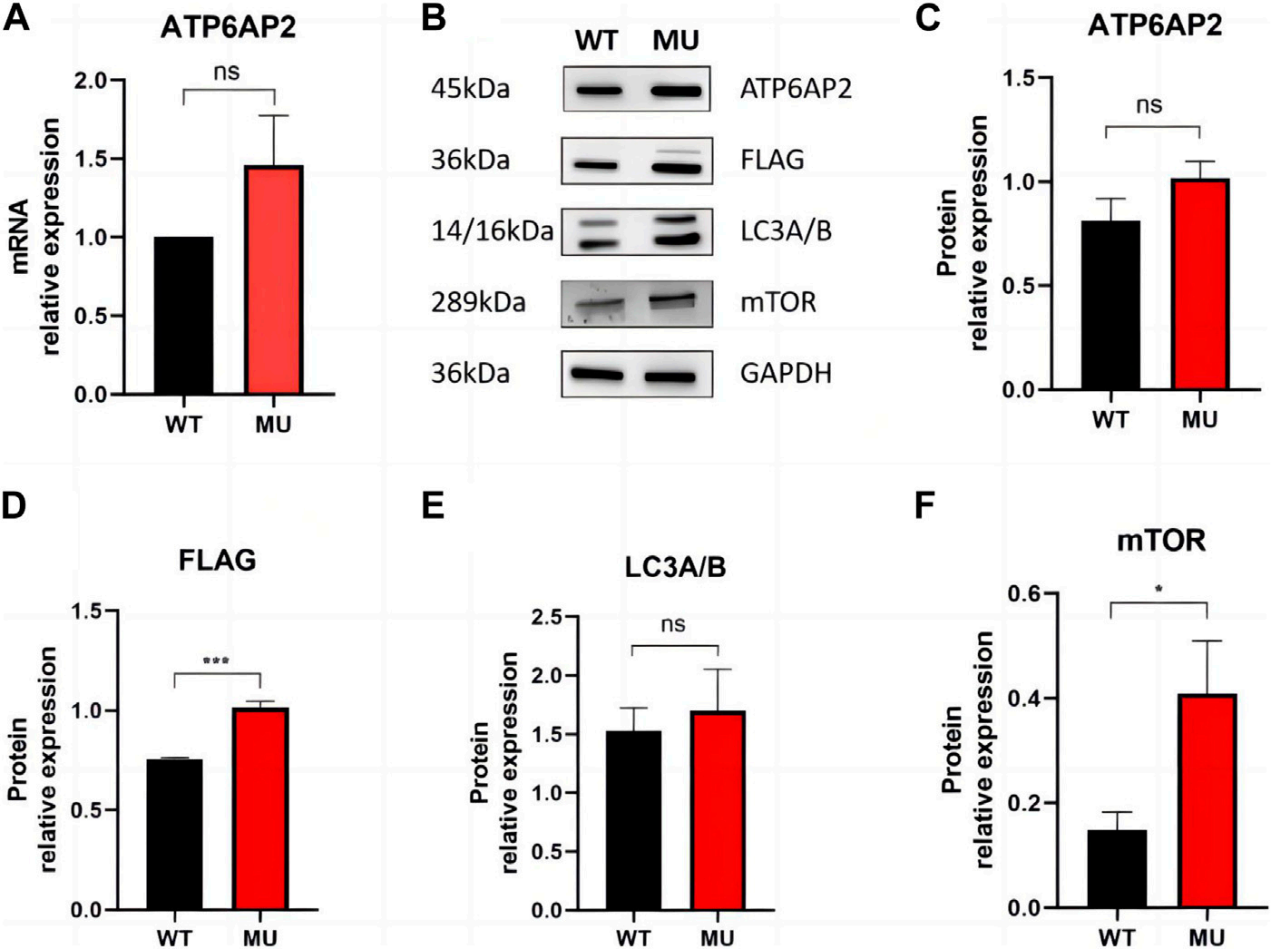
Expression of ATP6AP2, FLAG, LC3A/B and mTOR in HEK293T cells harboring wild type and mutant. mRNA of *ATP6AP2* was slightly increased in mutant rather than in wild-type exogenous constructs, without significant difference **(A)**. Western blotting showed alterations of the proteins **(B)**. ATP6AP2 was increased without significant difference **(C)**, while the expression of FLAG confirmed the elevated level in the mutant **(D)**. LC3A/B was upregulated without significant difference **(E)**. But mTOR signaling was significantly upregulated **(F)**. ns represents no statistical significance, * represents *p* < 0.05, and *** represents *p* < 0.001.

In HEK293T cells, qRT-PCR showed mRNA of *ATP6AP2* was slightly increased in mutant rather than in wild type exogenous constructs (Figure 3A), but the difference was not statistically significant (*p* = 0.1000). Western blotting (Figure 3B) revealed that ATP6AP2 protein (Figure 3C) was also increased without significant difference (*p* = 0.0563). Whereas the expression of FLAG (Figure 3D) tagged in the N-terminal of ATP6AP2 confirmed the elevated level in mutant cells (*p* < 0.001). The autophagosome marker, LC3A/B (Figure 3E), was upregulated without significant difference (*p* = 0.5053). And mTOR signaling (Figure 3F) was significantly upregulated (*p* < 0.05).

### Metabolomics signature of ATP6AP2^Gly62Glu^ mutant

**FIGURE 4:**
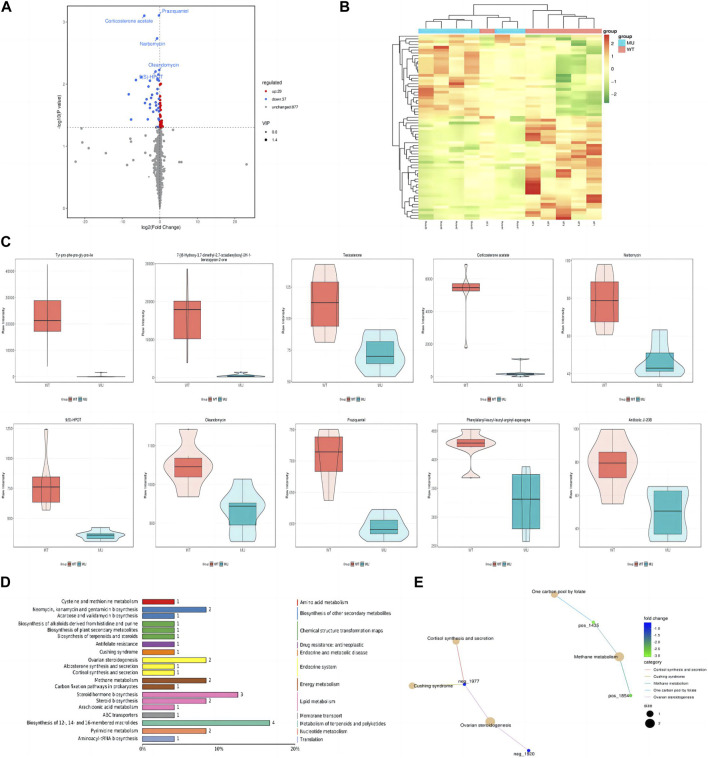
Signature of metabolomics. The volcano plot exhibited that metabolite downregulation was more pronounced **(A)**. Difference in metabolism was depicted in the heat map as well **(B)**. The violin diagram shows the top 10 metabolites with the smallest *p*-value **(C)**. The KEGG database was applied to annotate the differential metabolites, and the top 20 entries with the most differential metabolites in the pathway were selected **(D)**. The enrichment netplot shows the interaction and connection **(E)**.

In HEK293T cells, a total of 5,225 peaks were detected, of which 943 metabolites were annotated. Compared with the wild-type group, there were 66 differential metabolites, containing 29 ups and 37 downs. As shown by volcano plot, metabolite downregulation was more pronounced (Figure 4A). The difference in metabolism between wild types and mutants is depicted in a heat map as well (Figure 4B). It was observed that the top 10 metabolites with the smallest *p*-value were significantly lower in ATP6AP2^Gly62Glu^ than in ATP6AP2^WT^: tyr-pro-phe-pro-gly-pro-ile, 7-[(6-Hydroxy-3,7-dimethyl-2,7-octadienyl)oxy]-2H-1-benzopyran-2-one, testosterone, corticosterone acetate, narbomycin, 9(S)-HPOT, oleandomycin, praziquantel, phenylalanyl-leucyl-leucyl-arginyl-asparagine, and antibiotic JI-20B (Figure 4C). In organisms, different metabolites interact to create various pathways. The KEGG database was used to annotate the differential metabolites, and the top 20 entries with the most differential metabolites in the pathway were selected. A summary bar chart and annotation category are illustrated in Figure 4D. The most involved metabolic pathway was steroid hormone biosynthesis, steroid biosynthesis, and arachidonic acid metabolism in lipid metabolism. Metabolism of terpenoids and polyketides as well as the endocrine system were involved. The enrichment netplot shows similar results (Figure 4E).

## Discussion

ATP6AP2-CDG is a rare glycosylation defective disorder, which results from mutations in the X-linked *ATP6AP2.* To date, only three European (Germany and Portugal) patients with ATP6AP2-CDG have been described (Rujano et al., 2017). Incorporating the current patient, there are four reported cases of ATP6AP2-CDG, whose main manifestations were listed in Table 2. European patients were all males (3/3) with a current age range of 10 months–21 years, two of whom were from the same family. Patients had disease onset since birth (2/3). Clinical manifestations included neonatal icterus (2/3), mild to moderate or pronounced cutis laxa (3/3), mild cognitive impairment (2/3), ataxic gait (1/3), and dysmorphic features (2/3). The gender, age, onset symptoms, and main presentations of the Chinese patient were consistent with the Europeans. In addition, he presented with growth retardation, cardiomegaly, anemia, and thrombocytopenia. For laboratory test results, European patients presented high fluctuating levels of alanine aminotransferase and aspartate aminotransferase (3/3) as well as elevated cholesterol and LDL cholesterol (2/3). Reduced coagulation factors, prolonged activated partial thromboplastin time and prothrombin time, increased D-dimer and international normalized ratio, and decreased fibrinogen and prothrombin time activity indicated coagulation dysfunction in them (3/3). The Chinese patient also had hypertransaminasemia, hypercholesterolemia, and coagulopathy but with a low level of serum ceruloplasmin. The European patients presented with immunodeficiency (3/3), suffering recurrent infections; this was not present in the Chinese patient. To understand the severity of liver damage, European patients underwent histological examination of the liver (2/3). Morphological features were consistent with micronodular hepatic cirrhosis (2/2), accompanied by moderate macro- and microvesicular steatosis (1/2). Transmission electron microscopy revealed lipid deposition and increased vacuolar structures inside hepatocytes (1/3). There were no other findings on liver histology in the Chinese patient. Generally, ATP6AP2-CDG mainly affects the liver and the nervous system, though the immune and lymphatic hematopoietic system may be involved as well. WES demonstrated hemizygous missense mutations in *ATP6AP2* in European patients (3/3), including c.212G>A (p.Arg71His) and c.293C>T (p.Leu98Ser). The Chinese patient had a hemizygous missense mutation in novel c.185G>A (p.Gly62Glu) in *ATP6AP2*. *ATP6AP2* (NM_005765, ENST00000636580.2) is located on chromosome Xp11.4 (chrX: 40,580,970-40,606,848) and contains nine exons encoding a protein of 350 amino acids. The encoded ATP6AP2 [ATPase H^+^ transporting accessory protein 2, also known as the (pro) renin receptor] interacts with renin or prorenin on the cell surface to affect protein activity in one way, and acts as a part of the proton pump associated with adenosine triphosphatase (ATPase) in the other way (Wendling et al., 2017; Guida et al., 2018). Acidification of intracellular compartments, secondary active transport, energy conservation, and cellular pH regulation are all important functions of proton-translocating ATPases (Nelson et al., 2000; Scarborough, 2000; Tomashek and Brusilow, 2000). ATPases can be divided into the F, P, and V classes. The transmembrane proton-conducting sector V0 and the extramembrane catalytic sector V1 are both present in vacuolar (V-type) ATPases (Forgac, 2007). It has been discovered that ATP6AP2 is connected to the V0 sector of the V-type ATPases (Guida et al., 2018). Patients with mutation of c.185G>A (p.Gly62Glu), c.212G>A (p.Arg71His), or c.293C>T (p.Leu98Ser) presented a glycosylation disorder with predominant liver disease, immunodeficiency, and developmental delays. ATP6AP2 ablation affects multiple organs in adult mice (Wendling et al., 2017), providing the supporting basis. The cardiomegaly in the Chinese patient might be associated with the ATP6AP2 variation, indicating the importance of cardiac evaluation and follow-up in these patients. In cardiomyocytes, ATP6AP2 is essential for vacuolar H^+^-ATPase assembly, and the loss-of-function could result in lethal heart failure (Kinouchi et al., 2010; Li et al., 2022). Mutations in TMEM199 (Vma12) and CCDC115 (Vma22), another two human homologues of yeast, could result in the similar main clinical phenotypes (Jansen et al., 2016a; Jansen et al., 2016b; Fang et al., 2022). Therefore, V0 mis-assembly is speculated to be the common pathogenic process in these syndromes. In the previous study (Rujano et al., 2017), the mRNA level of the mutant Arg71His not Leu98Ser was elevated, while the level of both mutant proteins was decreased. A cycloheximide chase assay uncovered a shorter half-life in both mutant proteins. It indicated that disease-associated mutations targeted ATP6AP2 for degradation. In addition, the p.Leu98Ser mutation led to developmental defects, protein instability, and lipid homeostasis impairment in functional experiments. The previous study also found that the missense mutations impaired the interaction between ATP6AP2 and other V-ATPase assembly factors, which generally formed a complex (Rujano et al., 2017). For the current identified p.Gly62Glu mutation, immunohistochemistry detected no obvious expression changes of ATP6AP2 in the patient’s liver sample. Then we introduced an exogenous mutant into HEK293T cells, which did not show increased mRNA or protein expression of ATP6AP2 in Gly62Glu mutant cells with respect to wild type, indicating that c.185G>A (p.Gly62Glu) in *ATP6AP2* had no influence on its expression. Our predicted protein model displayed that the non-polar aliphatic amino acid glycine was substituted by acidic amino acid glutamic acid with altered polar contact with around Arg31 and Trp60. Because there is no side chain on the glycine in the wild type, the protein is more flexible at this point. While the variant residue glutamic acid has a negatively charged side chain, making it hydrophilic to prefer the surface of the protein to its interior. It suggests that the p.Gly62Glu mutation may have different effects on protein function, though there is a lack of direct evidence. Further research is needed to investigate the influence and association with the patient’s presentation.

**TABLE 2:** Main findings of the four patients with ATP6AP2-CDG.

Patient	Patient 1	Patient 2	Patient 3	Patient 4
	Family 1			
Reference	Rujano et al. (2017)	Rujano et al. (2017)	Rujano et al. (2017)	Current study
Gender	Male	Male	Male	Male
Current age	21 years	10 months	17 years	21 months
Onset of symptoms	Directly after birth	Directly after birth	5 months	Directly after birth
Country of origin	Germany	Germany	Portugal	China
Neonatal icterus	Absent	Present	Present	Present
Cutis laxa	Pronounced, improvement over time	Pronounced	Mild to moderate	Pronounced, improvement after liver transplantation
Developmental abnormalities	Ataxic gait, mild cognitive impairment	Normal	Mild cognitive impairment	Growth retardation
Other manifestation	Low-set ears, micrognathia, a flat and wide-set chest, laterally facing nipples, and hypospadias	NA	Mild dysmorphic features	Cardiomegaly, anemia and thrombocytopenia
Elevated transaminases	ALT 59 IU/L; AST 92–135 IU/L	AST 100–160 IU/L	ALT 51 IU/L; AST 61 IU/L	ALT 15.22–68.72 IU/L; AST 27.07–241.00 IU/L
Serum lipids	LDL cholesterol: 149 (reference: 50-130 md/dL)	Cholesterol: 254 (reference: 81–147 mg/dL)	Normal	Total cholesterol: 7.24 (reference: 3.1–5.2 mmol/L) LDL-cholesterol: 5.12 (reference: 1.30–3.90 mmol/L)
Ceruloplasmin (0.22–0.58 g/L)	NA	NA	NA	<0.02–0.17
Coagulation parameters	Low factor XI and free Protein S	Low factors Ⅱ, Ⅴ, Ⅶ, Ⅸ, and XI	Low factor Ⅴ and Ⅶ	Prolonged APTT and PT, increased D-dimer and INR, and decreased fibrinogen and PTA
Infection	Recurring pulmonary and upper respiratory tract infections	Recurring upper respiratory tract infections	Recurrent sepsis and peritonitis	Absent
Liver histology	NA	Diffuse micronodular hepatic cirrhosis	Micronodular hepatic cirrhosis with moderate macro- and microvesicular steatosis	Micronodular hepatic cirrhosis with moderate macro- and microvesicular steatosis
Hepatic ultrastructure	NA	Accumulations of autolysosomes	NA	NA
Zygosity	Hemizygous	Hemizygous	Hemizygous	Hemizygous
Mutation sites	c.212G>A	c.212G>A	c.293C>T	c.185G>A
Amino acid change	p.Arg71His	p.Arg71His	p.Leu98Ser	p.Gly62Glu
Glycosylation studies	Abnormal N-glycosylation	Abnormal N-glycosylation	Abnormal N-glycosylation	NA
Treatment	IgG substitution for immunodeficiency	NA	Weekly subcutaneous Ig for immunodeficiency	Living related liver transplantation for chronic liver failure

NA: not available.

Autophagy is a lysosomal breakdown process that controls the homeostasis of proteins and organelles, and it is essential for maintaining a healthy liver and regulating hepatic physiology and metabolism (Ke, 2019). Since ATP6AP2-CDG causes liver disease mainly, autophagy flux was explored in those patients. In Rujano et al. (2017) study, autophagosome markers p62 and Atg8a/LC3 were increased in ATP6AP2^Leu98Ser^ animals, suggesting increased autophagosome formation and/or decreased autophagic degradation. The enlarged autophagosomes were observed with undegraded content (lipid droplets) under electron microscopy. Meanwhile, LC3A/B was not elevated in the current patient’s liver but increased in the HEK293T cells with a p.Gly62Glu mutation. As the last step of autophagic breakdown is typically dependent on V-ATPase-mediated acidification, the decrease in V-ATPase activity caused by ATP6AP2 mutations induces an autophagy defect. Notably, it is well known that the mTOR pathway is essential for the regulation of autophagy (Wang et al., 2019). mTOR recruits two different signaling complexes, mTOR complex 1 (mTORC1) and mTOR complex 2 (mTORC2). In ATP6AP2^Leu98Ser^ animals, mTOR signaling was reduced in agreement with the proposed role of the V-ATPase in mTORC1 activation at the lysosomal-membrane (Rujano et al., 2017). The mTORC1 has been clarified to negatively regulate autophagy in multiple pathways (Kim et al., 2011; Martina et al., 2012; Park et al., 2016). However, it is unclear how exactly mTORC2 controls autophagy. The current finding of dysregulated autophagy and mTOR signaling seems to be consistent with previous knowledge.

The deficiency caused by c.212G>A (p.Arg71His) and c.293C>T (p.Leu98Ser) in *ATP6AP2* was proved to result in hypoglycosylation of serum proteins in patients and mice (Rujano et al., 2017). The prior patients showed aberrant tetrasialotransferrin and tri-, di-, and monosialotransferrin glycosylation patterns, which indicated N-Glycosylation disorder. The mice model treated with adeno-CRE induced liver-specific acute ATP6AP2 decrease; consequently, the liver enzymes and lipid parameters were elevated, which illustrated that ATP6AP2 defect could lead to liver damage and metabolic abnormalities. Transferrin evaluation, the main biomarker of CDG, was not possible in this patient because of the liver transplantation. In this study, the metabolomics signature of ATP6AP^Gly62Glu^ mutant introduced into HEK293T cells elucidated that most metabolites were downregulated and involved in the pathway of lipid metabolism. The patient carrying c.185G>A (p.Gly62Glu) presented with severe liver impairment (cirrhosis and steatosis) with elevated serum lipids before liver transplantation. Considering the obvious growth retardation as well, we speculate that the patient has metabolic dysfunction of lipids, which accumulate in the liver and cannot be transported, utilized, and energy supplied normally. Nevertheless, there were limitations of untargeted metabolomics and metabolomics itself, thus abnormalities in glucose metabolism were not detected. In addition, as the patient-derived live cells were not available, the metabolomics in this study is not sufficient to recapitulate the patient’s real situation, which will facilitate the initiation of our further study.

Optional treatments are limited in CDGs, including substrate supplementation, cofactor supplementation, pharmaceutical chaperons, non-causative, and other treatment (Park and Marquardt, 2021). In addition, organ transplantation, such as liver transplantation for end-stage cirrhosis, was recommended recently. Successful liver transplantation was reported in ATP6AP1-CDG, MPI-CDG, and PMM2-CDG (Janssen et al., 2014; Alharbi et al., 2023; Semenova et al., 2023; Tahata et al., 2023). After liver transplantation in the patients, not only the specific clinical presentation was improved, but the abnormal glycosylation was also restored. It demonstrated that liver transplantation could be a curative option for certain CDG patients with late course of hepatopathy. However, no treatment and prognosis has been described for ATP6AP2-CDG previously. The Chinese patient was treated with albumin and fibrinogen infusion, oral administration of D-galactose, UDCA, and vitamin D/AD/E/K1 supplements. Yet liver transplantation was performed eventually because of chronic liver failure with decompensated cirrhosis at a high death risk. Liver function parameters gradually normalized, and cutis laxa also improved post transplantation. These observations illustrated the current effectiveness of liver transplantation. Although the patient presented no significant disease progression during 1 year follow-up, long-term surveillance is still necessary. Subsequent *in vitro* and *in vivo* trials will be conducted to further investigate the pathogenicity of the novel c.185G>A (p.Gly62Glu) in *ATP6AP2* and potentially effective therapeutic strategies.

## Conclusion

This work presents the first Chinese patient with additional growth retardation, cardiomegaly, anemia and thrombocytopenia, and the c.185G>A (p.Gly62Glu) mutation in *ATP6AP2* exon 3, expanding the phenotypic and genotypic profile of ATP6AP2-CDG. The missense mutation dysregulated autophagy and mTOR signaling. The majority of metabolites were downregulated and implicated in the pathway of lipid metabolism in the exogenous Gly62Glu mutant. It establishes the groundwork for future investigations into the pathogenesis of ATP6AP2-CDG.

## Data Availability

The data presented in this study is included in the article/supplementary material. The datasets are not readily available due to privacy restrictions, further inquiries should be directed to the corresponding authors.
